# The 6-Minute Walk Test and Anthropometric Characteristics as Assessment Tools in Patients with Obstructive Sleep Apnea Syndrome. A Preliminary Report during the Pandemic

**DOI:** 10.3390/jpm11060563

**Published:** 2021-06-16

**Authors:** Vasileios T. Stavrou, George D. Vavougios, Kyriaki Astara, Dimitra I. Siachpazidou, Eirini Papayianni, Konstantinos I. Gourgoulianis

**Affiliations:** 1 Laboratory of Cardio-Pulmonary Testing and Pulmonary Rehabilitation, Department of Respiratory Medicine, Faculty of Medicine, University of Thessaly, 41110 Larissa, Greece; kyriakiastarara@gmail.com (K.A.); eirinipapayianni@gmail.com (E.P.); kgourg@uth.gr (K.I.G.); 2 Department of Respiratory Medicine, Faculty of Medicine, University of Thessaly, 41110 Larissa, Greece; dantevavougios@hotmail.com (G.D.V.); sidimi@windowslive.com (D.I.S.); 3 Department of Computer Science and Telecommunications, University of Thessaly, 35131 Lamia, Greece; 4 Department of Neurology, Athens Naval Hospital, 11521 Athens, Greece

**Keywords:** sleep disorders, 6-min walk test, body composition, anthropometry, oxidative stress

## Abstract

Patients with obstructive sleep apnea syndrome (OSAS) exhibit low cardio-fitness impact, attributed to fragmented sleep architecture and associated pathophysiological sequelae. The purpose of our study was to investigate fitness indicators during 6-min walk test (6MWT) and oxidative stress markers in apnea-hypopnea index (AHI) in OSAS patients stratified by severity. A total of 37 newly diagnosed patients, comorbidity-free, were divided into two groups: (Moderate OSAS (*n* = 12), defined as ≥ 15 AHI < 30 events per hour; Age: 50.7 ± 7.2 years, BMI: 32.5 ± 4.0 kg/m^2^ vs. Severe OSAS (*n* = 25), defined as AHΙ ≥ 30 events per hour; Age: 46.3 ± 10.4 years, BMI: 33.3 ± 7.9 kg/m^2^). Measurements included demographics, anthropometric characteristics, body composition, blood sampling for reactive oxygen metabolites’ levels (d-ROM) and plasma antioxidant capacity (PAT), and followed by a 6MWT. AHI was significantly associated with d-ROMs levels, chest circumference in maximal inhalation and exhalation (Δchest), neck circumference, as well as 6MWT-derived indices. In conclusion, our study determines bidirectional interrelationships between OSAS severity and anthropometrics, body composition, and fitness metrics. These findings indicate that the impact of OSAS should be evaluated well beyond polysomnography-derived parameters.

## 1. Introduction

Obstructive sleep apnea syndrome (OSAS) is characterized by recurrent episodes of partial or complete collapse of the upper airway during sleep, despite continuing respiratory efforts [1]. The Apnea–Hypopnea Index (AHI) is utilized to assess the severity of the syndrome among patients [2] and its severity has been associated with several comorbidity phenotypes [3]. The AHI, aligned with desaturation index (DI), reflects one of the most prominent detrimental consequence of OSAS, namely, intermittent nocturnal hypoxia. Intermittent hypoxia is responsible for increasing the risk of cardiovascular diseases’, prompted by endothelial dysfunction and the production of reactive oxygen species (ROS), leading to several pathophysiologic features that accompany OSAS [4]. Τhe chronicity of this phenomenon enriches the clinical manifestation of OSAS with daytime clinical entities, such as arterial hypertension and neurocognitive impairment; a cascade perpetuated by several factors, like age, body mass index (BMI), and male gender [5,6]. Due to the principal role of oxidative stress in orchestrating the course of OSAS, efforts have been made to nominate the possible biomarkers for predicting the severity and the comorbidity risk in OSAS patients [7].

Among anthropometric factors, BMI and other body composition indexes play a pivotal role in OSAS, as weight gain has been associated with exacerbation of apneic episodes [8]. Neck circumference assesses cervical obesity, which has been correlated with upper airway collapsibility, making it an accurate predictor of the severity of OSAS [9]. Body fat composition is related to oxidative stress, since adipose tissue contains endocrine properties, triggering the production of ROS on an inflammatory substrate [10]. In the context of OSAS, this relationship becomes reciprocal as OSAS patients manifest both psychological and cardiorespiratory intolerance to exercise [11]. Due to the multisystemic effects of sleep disturbances, patients exhibit diminished aerobic capacity, as evaluated by cardiopulmonary exercise testing [12]. Hence, fitness indicators could potentially serve as additional predictors for OSAS severity.

The purpose of our study was to investigate associations and differences between fitness indicators captured by the 6-min walk test (6MWT), anthropometrics, body composition, oxidative stress markers, and OSAS severity, as determined via the Apnea–Hypopnea Index (AHI).

## 2. Materials and Methods

### 2.1. Study Population

A total of 37 newly diagnosed, comorbidity free OSAS patients, were included in our study. Inclusion criteria were patients presenting ≥15 events/h on the Apnea–Hypopnea Index, non-smokers, age between ≥20-to-≤65 years old, sleep duration ≥300 min during polysomnography study (PSG), without recent injury (i.e., tendinitis, muscles injuries) [13], daily physical strain due to working ≥3 h/day [14], and weekly exercise ≥100 min per week with a heart rate ≥60 % of maximum [15]. 

Exclusion criteria were BMI ≥ 40 kg/m^2^ and comorbidities such as mellitus diabetes, heart failure, chronic obstructive pulmonary disease and other respiratory illness, peripheral vascular disease limiting exercise, myocardial infarction within the previous month, severe uncontrolled arrhythmia, severe and uncontrolled hypertension, severe aortic stenosis, renal failure, anemia, mental illness, and any form of musculoskeletal disability which could impair maximum exercise capacity, as per previously described protocols from our group [16].

#### Study Ethics

The study was approved by the Institutional Ethics Committee of University Hospital of Larissa (Ethical Committee Approval Number: 51114/04-11-19) and all participants submitted written consent according to the Helsinki declaration for use in Human subjects [17] and personal data (37/A/29-8-2019 and EC 2016/679) according to European Parliament and of the Council of the European Union.

### 2.2. Measurements

#### 2.2.1. Medical History, Anthropometrics, and Questionnaires 

Recorded data included complete medical history, demographics, anthropometrics [i.e., body height, chest circumference in maximal inhalation and exhalation, (Δchest), waist–hip ratio (WHR)], body mass and body composition, and estimated the resting metabolic rate, total body water (Tanita MC-980, Tokyo, Japan), body mass index (BMI), and body surface area [18]. The Pittsburgh Sleep Quality Index (PSQI) [19] and Epworth Sleepiness Scale (ESS) [20] questionnaires were administered prior to the 6MWT. Historical data from previous polysomnography were also available for each patient. Sleep staging was performed via manual scoring according to the Rechstaschaffen and Kales [21]. Stages 1 to 3 represent non-rapid eye movement (NREM) sleep, whereas stage 4 represents rapid eye movement sleep (REM). Pulmonary function parameters (FEV_1_: forced expiratory volume in 1st sec, FVC: forced vital capacity, Master Screen-CPX, VIASYS HealthCare, Hochberg, Germany) [22] were recorded prior to data collected.

#### 2.2.2. Oxidative Stress Markers 

Blood sampling for oxidative stress measurement was performed 20 min before 6MWT. Initially, a 10 mL sample was of peripheral venous blood was collected from each patient at 08.30 a.m., having fasted the previous night. Measurements included the determination of reactive oxygen metabolites’ levels (d-ROMs test) and the plasma antioxidant capacity (PAT test) (free radical analytical system, FRAS5, Parma, Italy). Conceptually, the d-ROMs test provided an estimate of oxidative burden, whereas the PAT quantified water-soluble antioxidant contents within the same plasma sample.

#### 2.2.3. The 6 Min Walk Test 

Measurements during 6MWT [23] included arterial O_2_ saturation (SpO_2_) and heart rate (HR) (Ri-fox, Riester, Germany) at the following timepoints: baseline, every 1 min of test and at the 1st min of recovery. Blood pressure (BP, Mac, Tokyo, Japan) and self-assessed lower extremity fatigue with dyspnea Borg Scale CR10 [24] were recorded at the following timepoints: baseline, end of test and at the 1st min of recovery.

Additional measurements captured included total distance and peak O_2_ uptake [25] and metabolic equivalent (METs = peak O_2_ uptake (mL/min/kg)/3.5).

All sessions were performed in Laboratory of Cardio-Pulmonary Testing and Pulmonary Rehabilitation (University of Thessaly), with environmental temperature at 22 ± 1 °C and humidity 45 ± 3%. The evaluation was made between 08:30 a.m. to 11:00 a.m.

### 2.3. Statistical Analysis

Several established cut-off points were used for data stratification. The cut-off points for oxidative stress (d-ROMs: 320 Carr.U. and PAT: 2200 U. cor.) and visceral fat (12 score) were classified according to the manufacturer’s instructions for distinguishing between healthy and border condition adults. 6MWT was set as a cross-sectional point that was 85% of the predicted value, according Ross formula [25], for distinguishing between normal and low cardiopulmonary function [26]. The neck circumference cut-off, for distinguishing between low and high priority for the overnight sleep study for suspect OSAS, was set as 40 cm [27]. An appropriate cut-off for Δchest has not be universally established, and reported associations include smoking habit, age, gender, and the co-existence of respiratory disorders [28]. Thus, a 5% difference between maximal inhalation and maximal exhalation was set as the cut off for distinguishing between well and less well enlargement of the chest cavity [29]. For continuous variables, data normality was assessed via the Kolmogorov-Smirnov test. Relationships between continuous variables were assessed via the Spearman’s Rho and Pearson’s R correlation coefficients for non-parametric and parametric variables, correspondingly. Differences between AHI strata were assessed via the independent samples *t*-test or Mann–Whitney U-test where appropriate. For all aforementioned tests, a *p*-value < 0.05 was considered statistically significant. Continuous data are presented as mean ± standard deviation (Mean ± SD). All analyses were performed via the SPSS 25 statistical package (SPSS Inc., Chicago, IL, USA).

## 3. Results

**Figure 1:**
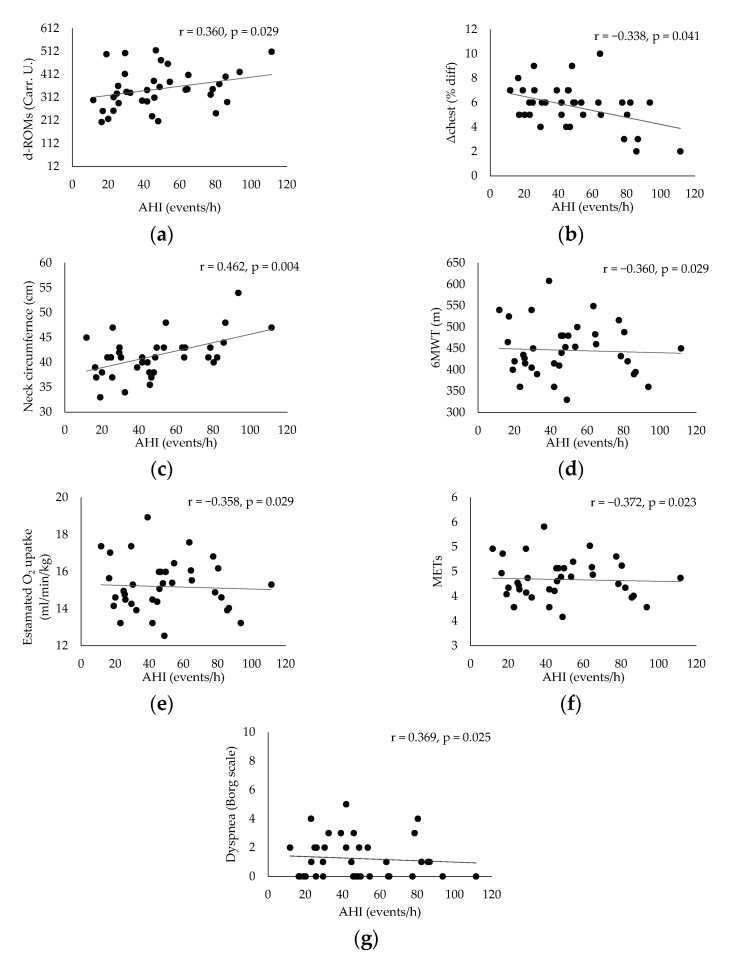
Correlation analysis results between apnea hypopnea index (AHI) and reactive oxygen metabolites’ levels (d-ROM) (**a**), chest circumference difference between maximal inhalation and exhalation (Δchest) (**b**), neck circumference (**c**), meters during 6 min walk test (6MWT) (**d**), estimated oxygen uptake (VO_2_) (**e**), metabolic equivalent (MET) (**f**) and dyspnea at the end of 6MWT (**g**).

**Figure 2:**
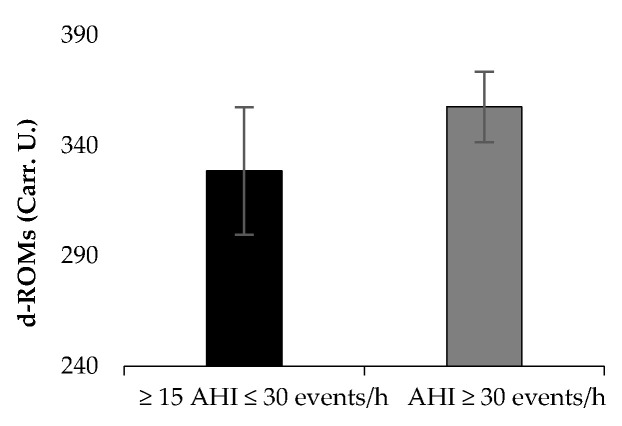
Oxidative stress marker on reactive oxygen metabolites’ levels (d-ROM) between groups.

**Figure 3:**
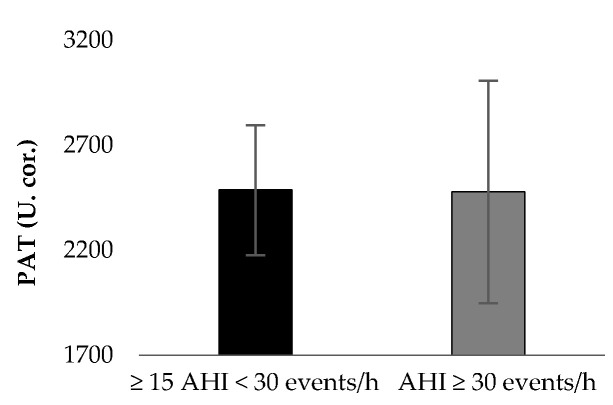
Oxidative stress marker on plasma antioxidant capacity (PAT) between groups.

**Figure 4:**
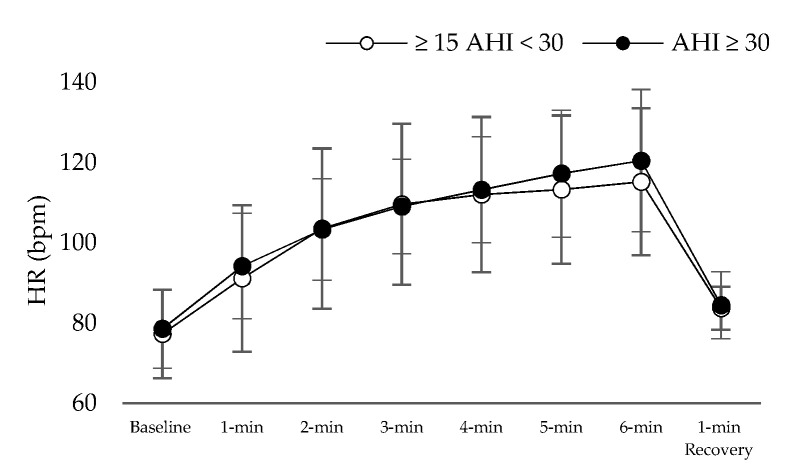
Heart rate (HR) alteration during 6 min walk test.

**Figure 5:**
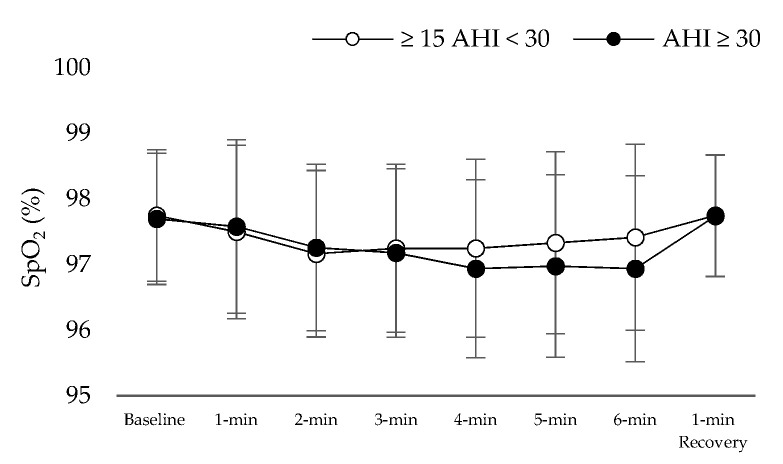
Oxygen saturation (SpO_2_) alteration during 6 min walk test.

**Figure 6:**
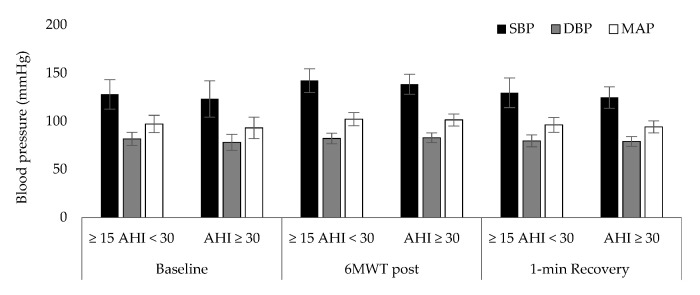
Blood pressure [(Systolic blood pressure (SBP), diastolic blood pressure (DBP), and mean arterial pressure (MAP)] alteration during 6 min walk test (6MWT).

Table 1 presents the results of patient characteristics between two severity strata: Moderate (defined as 15 ≥ AHI > 30 events per hour) vs. Severe OSAS (defined as AHI ≥ 30 events per hour). Statistically significant correlation were detected between AHI (Figure 1) and d-ROMs levels (r = 0.360, *p* = 0.029), Δchest (r = −0.338, *p* = 0.041), neck circumference (r = 0.462, *p* = 0.004,) and parameters during 6MWT (Meters: r = −0.360, *p* = 0.029, estimated O_2_ uptake: r = −0.358, *p* = 0.029, METs: r = −0.372, *p* = 0.023, Dyspnea_Borg scale_ at the end of 6MWT: r = 0.369, *p* = 0.025). In Table 2, the cut-off points of studied variables are presented with respect to variables of polysomnography. The variabilities dROMs (≥15AHI < 30 events/h: 328.5 ± 28.9 versus AHΙ ≥ 30 events/h: 357.7 ± 15.9 U carr, *p* = 0.342, Figure 2), PAT (≥15AHI < 30 events/h: 2486.7 ± 309.7 versus AHΙ ≥ 30 events/h: 2477.3 ± 530.6 U cor, *p* = 0.955, Figure 3), anthropometric characteristics and body composition (Table 1) and fitness indicators during 6MWT (Figure 4, Figure 5 and Figure 6), distance (≥15AHI < 30 events/h: 460.6 ± 64.9 versus AHΙ ≥ 30 events/h: 438.3 ± 60.1 m, *p* = 0.311; ≥15AHI < 30 events/h: 80.8 ± 13.7 versus AHΙ ≥ 30 events/h: 74.1 ± 14.3 % of predicted, *p* = 0.183), estimated O_2_ uptake (≥15AHI < 30 events/h: 15.5 ± 1.5 versus AHΙ ≥ 30 events/h: 15.0 ± 1.4 mL/min/kg, *p* = 0.312) and metabolic equivalent (≥15AHI < 30 events/h: 4.5 ± 0.4 versus AHΙ ≥ 30 events/h: 4.3 ± 0.3 METs, *p* = 0.273) did not show differences between groups.

## 4. Discussion

In our study, OSAS severity was associated with worse indices for fitness, cognitive performance and increased oxidative burden. In our analyses, AHI was associated with anthropometric characteristics, such as neck circumference and Δchest. Previous studies have shown that increased BMI is associated with and increased prevalence of sleep disordered breathing [30] when BMI is adjusted via body composition parameters. As such, body composition would ideally be represented by a function of independent anthropometric quantified traits, indicative of local (i.e., visceral fat, peripheral subcutaneous fat, muscle etc.) and overall distribution (i.e., total body water) [31]. Thus, BMI adjusted via these variables describes body composition more accurately, and enables the recognition of latent relationships with AHI.

According to our study design, obesity class III patients were excluded. The vicious cycle of sleep apnea perpetuated by body composition can be extracted, independently of obesity related factors [32]. Notably, body composition parameters that are not directly associated with BMI have been shown to be independently associated with the pathophysiological mechanisms underlying OSAS. As an example, increased neck circumference hinders the airway patency, a phenomenon exacerbated during sleep [33]. A measurable consequence is increasing AHI, which, when prolonged, corresponds to hypercapnia and the development of respiratory acidosis [34] and oxidative burden [35]. These effects are the substrate of the observable diminished physical activity and proneness to breath shortness elicited via the 6MWT. The latter furthermore corresponds to less effective chest expansion and, hence, lower VO_2_ [36] and METs, independent of AHI severity [37]. Therefore, tools that assess fitness indicators, such as 6MWT, in conjunction with a more thorough recording of anthropometric and morphological characteristics could contribute to a more optimal characterization of OSAS patients and OSAS’ impact on their lives, while demystifying BMI.

One of the most important body composition parameters is adipose tissue. Adipose tissue encompasses several types, with visceral fat being the most clinically relevant as it is associated with more adverse effects compared to peripheral obesity [38], while there is an interrelationship between obesity and physical activity and cardiovascular diseases [39]. Moreover, visceral adipose tissue contributes to the activity of a chronic inflammatory substrate, which contributes to the underlying pathophysiology of sleep apnea [40]. Another finding of our study was that the drop in SaO_2_ indicators during sleep are affected by anthropometric indexes and visceral fat, aside from the expected association with AHI. This finding further stress the importance of both adipose tissue and anthropometrics in understanding patient related parameters that enhance the pathophysiology of sleep disordered breathing, and may implicate alveolar implication [16,41]. The combined drive of both alveolar and upper airway dysfunction would favor the gradual onset of a hypoxic milieu gradually establishes an oxidative environment where ROS production overcomes antioxidant compensation [42]. 

Another significant finding of our study was the association of severe OSAS with disrupted of sleep architecture. Specifically, was assessed via the correlation of deep non-rapid eye movement (NREM, Stage 3 and 4), of relatively young patients, with PAT test. Deep NREM is fundamental for several cognitive functions, like memory consolidation [43]. Previous findings from our group have indicated that cognitive performance is affected even in younger OSAS patients, even on the subclinical level [6]. Several factors may provide the pathophysiological substrate for these relationships. Sleep disturbances disrupt microarchitecture of sleep and, thus, the circadian-related clearance of proteins related to neurodegeneration [44]. Furthermore, intermittent hypoxia and hypercapnia hampers synaptic plasticity and neuronal survival, affecting by extent several cognitive networks [45]; within the same context, neuroinflammation and oxidative stress further exacerbate this noxious milieu, setting the stage for the emergence of neurodegeneration [46,47].

### Limitations and Strengths

The results of our study should be interpreted within the context of its limitations. Our study population was nested, and reports on patients were from a single University hospital. The University Hospital of Larisa is the referral tertiary institute for central Greece, including the mainland and several islands. Our study excluded by design class III obese patients, and, therefore, no insight can be gained from these patients. The inclusion of such a group however would require a different study design that could address the perturbations introduced by the interrelationships between class III obesity and breathing as a standalone clinical entity. Another important limitation of our study is that recruitment involved consecutive patients, and, therefore, could not provide equal size groups for, e.g., OSAS strata. We aimed to overcome this obstacle by selecting alternative stratification of our cohort using, i.e., oxidative stress and anthropometrics cut-offs, and assessing AHI differences instead.

## 5. Conclusions

The parameterization of the respiratory profile of sleep apnea through anthropometric characteristics that expand beyond body mass index might elucidate the pathophysiology of OSAS, in conjunction with body composition and fitness indicators. In this context, the 6MWT was an adequate substitute of cardiopulmonary exercise testing, when the latter was not available.

## Figures and Tables

**Table 1:** Patients characteristics. Data are expressed as mean ± standard deviation.

	Mean ± Sd (*n* = 37)	≥15 AHI < 30 Events/h, (*n* = 12)	AHΙ ≥ 30 Events/h, (*n* = 25)	*p* Value
Age, years	47.7 ± 9.6	50.7 ± 7.2	46.3 ± 10.4	0.194
Gender, *n* = M/F	31/6	9/3	22/3	-
AHI, events/h^−1^	48.0 ± 25.3	22.1 ± 5.4	60.4 ± 21.2	<0.001
Apnea, events/h^−1^	17.6 ± 20.0	3.1 ± 3.2	24.6 ± 21.0	0.001
Hypopnea, events/h^−1^	29.0 ± 14.0	19.0 ± 5.1	33.8 ± 14.4	0.002
Stage 1, %	4.0 ± 4.0	2.7 ± 0.8	4.7 ± 4.8	0.172
Stage 2, %	53.9 ± 15.0	51.8 ± 12.0	54.9 ± 16.3	0.569
Stage 3–4, %	11.0 ± 6.5	13.7 ± 6.7	9.7 ± 6.2	0.078
REM, %	9.7 ± 5.5	9.6 ± 5.1	9.8 ± 5.8	0.923
Desaturation Index, %	51.0 ± 28.8	22.7 ± 9.0	64.6 ± 24.7	<0.001
Mininum SaO_2_, % *^‡^*	75.6 ± 12.9	82.7 ± 7.4	72.3 ± 13.8	0.020
Average SaO_2_, % *^‡^*	87.6 ± 5.2	90.6 ± 1.7	86.1 ± 5.7	0.013
Duration SaO_2_ < 90%, min *^‡^*	40.9 ± 51.1	8.8 ± 13.5	56.3 ± 55.5	0.006
Body Mass Index, kg/m^2^	33.1 ± 6.8	32.5 ± 4.0	33.3 ± 7.9	0.749
Body fat, %	32.9 ± 9.1	35.2 ± 9.3	31.8 ± 9.1	0.303
Visceral fat, score	14.3 ± 5.7	14.3 ± 4.0	14.3 ± 6.4	0.979
Muscle mass, kg	34.4 ± 12.2	34.6 ± 13.6	34.2 ± 11.7	0.929
Body Surface Area, m^2^	2.4 ± 0.5	2.4 ± 0.3	2.4 ± 0.6	0.547
Lean Body Mass, %	78.6 ± 6.7	77.3 ± 5.6	79.3 ± 7.2	0.415
Total Body Water, %	49.5 ± 4.9	48.4 ± 4.3	50.0 ± 5.2	0.349
RMR, kcal/day	1932.2 ± 269.5	1879.0 ± 226.1	1957.8 ± 288.8	0.413
Neck circumference, cm	41.3 ± 4.1	40.3 ± 3.8	41.7 ± 4.3	0.340
WHR	0.9 ± 0.8	1.0 ± 0.1	0.9 ± 0.1	0.125
Δchest, %	5.6 ± 2.1	5.5 ± 2.3	5.9 ± 1.6	0.628
FEV_1_, % of predicted	94.9 ± 12.4	99.6 ± 6.1	92.7 ± 14.0	0.116
FVC, % of predicted	95.8 ± 10.6	100.3 ± 5.5	93.7 ± 11.8	0.077
PSQI, score	7.5 ± 5.2	8.9 ± 6.0	6.8 ± 4.7	0.268
ESS, score	7.7 ± 4.5	6.7 ± 2.5	8.1 ± 5.1	0.375

Abbreviations: AHI: Apnea–Hypopnea Index; ESS: Epworth Sleepiness Scale; F: Female; FEV_1_: forced expiratory volume in 1st sec; FVC: forced vital capacity; M: Male; PSQI: Pittsburg Sleep Quality Index; REM: Rapid Eye Movement; RMR: resting metabolic rate; WHR: Waist-hip ratio; Δchest: chest circumference difference between maximal inhalation and exhalation. *^‡^* Minimum, Average and Duration of SaO_2_ < 90% are polysomnography derived parameters.

**Table 2:** Comparisons between polysomnography-derived variables per subgroup. Data are presented as mean ± standard deviation.

	6MWT, 85% of Predicted		*p* Values	NC, 40 cm		*p* Values	Visceral Fat, 12 Score		*p* Values	Δchest, 5% Differences		*p* Values	d-ROMs, 320 Carr.U.		*p* Values	PAT, 2200 U. cor		*p* Values
*n*, < Versus >	*n* = 25	*n* = 12		*n* = 11	*n* = 26		*n* = 12	*n* = 25		*n* = 13	*n* = 24		*n* = 14	*n* = 23		*n* = 7	*n* = 30	
AHI, events/h	53.7 ± 27.4	36.3 ± 14.9	0.048	32.3 ± 13.1	54.6 ± 26.4	0.012	37.7 ± 14.0	52.9 ± 28.1	0.087	59.1 ± 29.9	41.9 ± 20.6	0.047	37.4 ± 23.0	54.5 ± 24.8	0.044	61.6 ± 30.7	44.8 ± 23.3	0.117
Apnea, events/h	22.9 ± 21.5	6.4 ± 9.7	0.016	9.2 ± 12.3	21.2 ± 21.7	0.097	12.6 ± 13.9	20.0 ± 22.2	0.295	21.3 ± 26.7	15.6 ± 15.4	0.416	11.9 ± 16.9	21.1 ± 21.2	0.179	32.5 ± 27.2	14.1 ± 16.6	0.026
Hypopnea, events/h	28.6 ± 15.2	29.8 ± 11.4	0.817	23.1 ± 8.7	31.5 ± 15.1	0.096	25.2 ± 8.5	30.9 ± 15.8	0.251	33.9 ± 17.4	26.4 ± 11.2	0.120	25.5 ± 15.3	31.2 ± 12.9	0.235	29.0 ± 14.9	29.0 ± 14.0	0.995
Stage 1, %	3.4 ± 2.1	5.4 ± 6.5	0.174	2.9 ± 1.5	4.5 ± 4.7	0.136	2.8 ± 1.7	4.6 ± 4.7	0.217	4.2 ± 2.7	3.9 ± 4.7	0.897	3.3 ± 2.5	4.5 ± 4.8	0.235	6.9 ± 8.1	3.4 ± 2.1	0.032
Stage 2, %	58.1 ± 12.4	45.1 ± 16.4	0.011	55.9 ± 11.3	52.9 ± 16.4	0.588	58.9 ± 11.2	51.4 ± 16.1	0.154	53.7 ± 15.6	53.9 ± 14.9	0.967	52.9 ± 13.4	54.5 ± 16.1	0.365	47.8 ± 19.0	55.3 ± 13.8	0.241
Stage 3–4, %	11.0 ± 6.4	11.0 ± 7.1	0.991	12.6 ± 7.4	10.4 ± 6.1	0.331	13.0 ± 6.9	10.1 ± 6.3	0.202	10.3 ± 6.2	11.5 ± 6.8	0.603	12.2 ± 7.0	10.4 ± 6.3	0.753	5.4 ± 5.7	12.4 ± 6.0	0.009
REM, %	9.7 ± 4.5	9.8 ± 7.5	0.983	10.5 ± 4.7	9.4 ± 5.9	0.574	11.4 ± 3.8	8.9 ± 6.1	0.197	9.9 ± 6.9	9.6 ± 4.8	0.897	10.7 ± 5.5	9.1 ± 5.5	0.420	9.3 ± 6.7	9.8 ± 5.3	0.802
Desaturation Index, %	55.4 ± 31.2	41.8 ± 21.2	0.986	31.5 ± 13.9	59.2 ± 29.6	0.006	36.5 ± 13.9	57.9 ± 31.6	0.032	67.0 ± 32.8	42.3 ± 22.6	0.010	37.1 ± 24.1	59.4 ± 28.5	0.398	69.2 ± 34.6	46.7 ± 26.1	0.062
Mininum SaO_2_, % *^‡^*	74.1 ± 14.7	78.9 ± 7.7	0.294	83.3 ± 7.6	72.4 ± 13.4	0.017	82.0 ± 8.7	72.6 ± 13.6	0.037	70.8 ± 15.3	78.2 ± 10.9	0.097	82.1 ± 8.2	71.7 ± 13.8	0.019	70.4 ± 14.6	76.9 ± 12.5	0.241
Average SaO_2_, % *^‡^*	87.0 ± 6.1	88.7 ± 2.4	0.382	90.3 ± 2.1	86.4 ± 5.7	0.038	89.9 ± 2.3	86.4 ± 5.9	0.014	85.5 ± 7.3	88.7 ± 3.4	0.082	±89.8	86.2 ± 6.0	0.016	85.1 ± 4.4	88.1 ± 5.3	0.176
Duration SaO_2_ < 90%, min *^‡^*	48.4 ± 58.5	25.3 ± 26.4	0.203	12.9 ± 18.2	52.7 ± 56.1	0.029	18.3 ± 21.2	51.8 ± 57.8	0.061	63.9 ± 68.5	28.5 ± 34.5	0.043	20.0 ± 19.3	53.6 ± 60.2	0.052	62.9 ± 49.2	35.8 ± 51.0	0.210

Abbreviations: 6MWT: 6-min walk test; AHI: Apnea Hypopnea Index; d-ROMs: reactive oxygen metabolites; *n*: number of patients; NC: neck circumference; PAT: plasma antioxidant capacity; REM: Rapid Eye Movement; Δchest: chest circumference difference between maximal inhalation and exhalation. *^‡^* Minimum, Average and Duration of SaO_2_ < 90% are polysomnography derived parameters.

## Data Availability

All data are available after request.
